# Oviplate: A Convenient and Space-Saving Method to Perform Individual Oviposition Assays in *Aedes aegypti*

**DOI:** 10.3390/insects9030103

**Published:** 2018-08-15

**Authors:** Rafaella Sayuri Ioshino, Danilo Oliveira Carvalho, Isabel Cristina Santos Marques, Ediane Saraiva Fernandes, Margareth Lara Capurro, André Luis Costa-da-Silva

**Affiliations:** Laboratório de Mosquitos Geneticamente Modificados, Departamento de Parasitologia, Instituto de Ciências Biomédicas, Universidade de São Paulo, São Paulo 05508-000, Brazil; carvalhodanilo85@gmail.com (D.O.C.); belmarquesusp@gmail.com (I.C.S.M.); edi.sfernandes@gmail.com (E.S.F.); mcapurro@icb.usp.br (M.L.C.)

**Keywords:** *Aedes aegypti*, oviposition, oviplate, fecundity assay, eggs, mosquito

## Abstract

*Aedes aegypti* is the principal vector of the urban arboviruses and the blood ingestion is important to produce the eggs in this species. To analyze the egg production in *Ae. aegypti*, researchers frequently use small cages or *Drosophila* vials to collect eggs from gravid females. Although it is affordable, the setup is time- and space-consuming, mainly when many mosquitoes need to be individually analyzed. This study presents an easy, cheap, and space-saving method to perform individual oviposition assays in *Ae. aegypti* using cell culture plates. This new method to access fecundity rate was named “oviplate”. The oviplates are setup with 12- or 24-well plates, distilled water and filter paper and they are 78 to 88% cheaper than the traditional *Drosophila* vial assay, respectively. Furthermore, to allocate 72 vitellogenic females in an insectary using *Drosophila* vial is necessary 4100 cm^3^ against 1400 cm^3^ and 700 cm^3^ when using 12- and 24-well plates, respectively. No statistical differences were found between the number of eggs laid in *Drosophila* vials and the oviplates, validating the method. The oviplate method is an affordable, and time- and space-efficient device, and it is simpler to perform individual fecundity analyses in *Ae. aegypti*.

## 1. Introduction

The importance of *Aedes aegypti* in spreading and sustainment of epidemics caused by Yellow fever (YFV) and Dengue (DENV) viruses in urbanized/anthropized environments is well described. Moreover, this species has also achieved a dramatic status as the main vector for emerging arthropod-borne viruses such as Zika (ZIKV) and Chikungunya (CHIKV) viruses in many tropical and subtropical regions worldwide, confirmed by laboratory and field studies [1,2,3,4,5,6].

Hematophagy is a relevant behavior in a mosquito vector’s life cycle. The blood meal is intrinsically associated with the pathogen transmission between invertebrate and vertebrate hosts [7]. Furthermore, it is also fundamental to trigger molecular and physiological processes as vitellogenesis and ovogenesis, culminating in egg production by the ovaries [8,9,10,11].

In *Aedes* genus, the vitellogenesis lasts for 72 h post-blood meal. After this time, females can lay their eggs on wet surfaces to complete the species’ life cycle [11]. The reproduction process in *Ae. aegypti* is one of the critical characteristics for its adaptation in the modified environments and the evolutionary development of desiccation-resistant eggshell is a trait providing species advantage over other mosquito species. This characteristic protects the embryonic development and keeps the embryo viable for several months (up to 12 months) under dry conditions [12,13].

The fecundity can be useful for researchers to explore and estimate the impacts of pathogen infections [14,15,16], resistance to insecticides as temephos [17], characterization of levels of vertical transmission [18,19,20,21,22], and verification of the sterility rate in *Ae*. *aegypti* males used for vector control purposes [23].

Regarding oviposition assays, the fecundity of *Aedes* can be analyzed by pooling the gravid females before egg laying starts. In this case, the total number of eggs or the average number of eggs laid per females can be estimated. To perform a pooled oviposition assay, the gravid females only need to be exposed to an artificial breeding site (a container halfway filled with water for example) at the same time. The same general procedure is performed for collecting eggs from mosquito colonies in laboratory scale. On the other hand, when the oviposition assay is applied to characterize variations between individuals or when more refined quantifications such as the real number of eggs per female are required, an individual oviposition analysis is the method of choice.

Different techniques such as individual holding cup [14], small cage [17], plastic cups [24], or tubes/*Drosophila* vials [23,25,26,27] are feasible for individual oviposition assays in mosquitoes. The last one was originally developed for *Drosophila* experiments [28,29,30,31], and adapted for mosquito oviposition assays (especially in *Aedes* genus). This method uses wet cotton covered with filter paper or only water-soaked funnel filter paper as a wet surface to allow *Aedes* females to lay eggs.

The individual oviposition assays are laborious and time-consuming, especially if a lot of insects need to be analyzed. Additionally, a considerable amount of shelving is required to keep them during oviposition, which may be particularly limiting if incubators are used.

In order to solve these limitations, we developed an easy, cheap, and space-saving method to perform individual oviposition assays in *Ae*. *aegypti* based on the use of cell culture plates. This straightforward method was named as “Oviplate” and uses 12- or 24-well plate with wet filter paper to perform the oviposition assays.

## 2. Material and Methods

### 2.1. Ethics Statement

This study used anaesthetized *Rattus norvegicus* (Wistar strain) to blood feed *Ae. aegypti* females. The use of these animals were carried out in accordance with the guidelines of the Ethical Principles for Experiment on Animals adopted by Sociedade Brasileira de Ciência de Animais de laboratório (SBCAL) and approved by the Institutional Ethics Review Committee (Comissão de Ética no Uso de Animais—CEUA-ICB-Universidade de São Paulo), protocol #186/2012.

### 2.2. Mosquitoes Rearing

*Ae*. *aegypti* Higgs strain were maintained in a BSL-2 insectary facility in Institute of Biomedical Sciences from University of São Paulo under the insectary conditions: 27 ± 1 °C, 75–80% relative humidity with 12/12-h (light/dark) photoperiod. *Ae. aegypti* immature forms (larvae) were fed with powdered fish food (Sera Vipan Premium, ref. # 00190 INT) and adults were maintained with a cotton wool moistened with 10% sucrose solution (*w*/*v*).

### 2.3. Blood-Feeding

Newly emerged, sex-sorted *Ae. aegypti* females and males were mated in a 3:1 proportion (females/males, respectively) and were maintained during 5–7 days on 10% sucrose solution (*w*/*v*). Pre-mated 5- to 7-day-old females were starved for 24 h and were fed on an anesthetized Wistar mouse (*Rattus norvegicus*) for 30 min. The females were CO_2_ anesthetized and fully engorged ones were sorted from non-engorged/partial engorged insects. Blood-fed females received ad libitum 60% sucrose solution (*w*/*v*) over five days post blood meal (PBM) when the individual oviposition assays were performed.

### 2.4. Drosophila Vial Set Up

To perform the *Drosophila* vial oviposition assay, 0.4 g of cotton was inserted at the bottom of a glass tube (85 mm high × 26 mm diameter). A rounded by cutting odorless blotting filter paper (Thermo Fisher Scientific, San Jose, CA, USA, # 05-714-4) with 2.5 cm diameter was pushed down inside the glass vial until it was snug against the cotton surface using a 15 mL conical centrifuge tube (with the lid screw thread part upside down). To moisten the surface (cotton and filter paper), 4.3 mL of tap water was added using a transfer pipette. *Drosophila* vials were plugged at the top by using 0.5 g of cotton. Gloves were used to assemble the vials to avoid human odor. The amount of cotton was weighted as described, but a small piece is sufficient for the setup.

### 2.5. Oviplate Set Up

**Figure 1:**
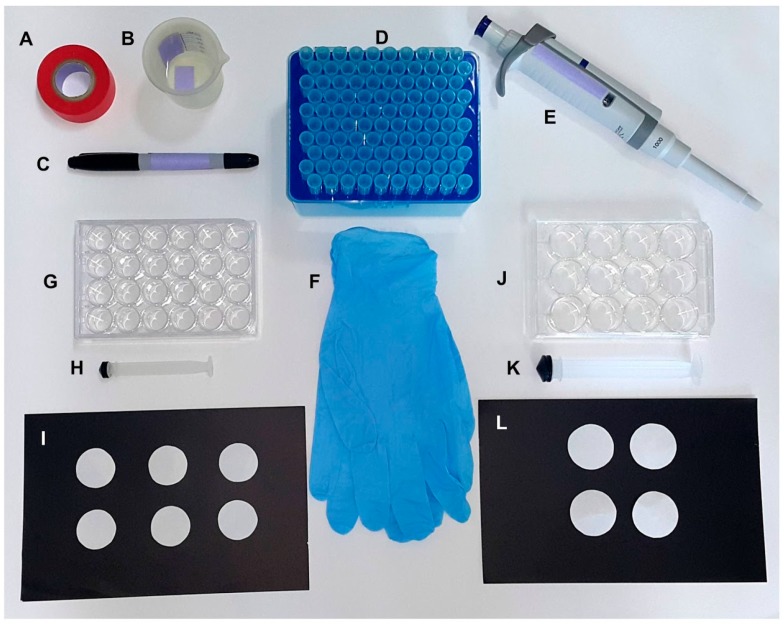
Materials required to set up the oviplates with 12- and 24-well cell culture plates. General materials for both oviplates: (**A**) tape; (**B**) tap water; (**C**) permanent marker; (**D**) 1.0 mL disposable tips; (**E**) p1000 pipette; (**F**) gloves. Specific materials for individual oviposition using 24 females: (**G**) 24-well cell culture plate; (**H**) 5.0 mL syringe plunger; (**I**) Circular filter paper with 2.0 cm diameter. Specific materials for individual oviposition using 12 females: (**J**) 12-well cell culture plate; (**K**) 20.0 mL syringe plunger; (**L**) Circular filter paper with 2.5 cm diameter.

The oviplates were assembled using 12- and 24-well cell culture plates. The materials used were as follows: colored labeling tape (Fisherbrand™), tap water, permanent marker (Sharpie^®^), disposable tips (1.0 mL), p1000 pipette, and gloves (Figure 1A–F). For the 12-wells oviplate, the same rounded odorless blotting filter paper (described above) was placed in wells of the cell culture plate (Corning™ Incorporated Costar™ number 3513) using a 20.0 mL syringe plunger (Figure 1J–L). The filter papers were wetted with 0.2 mL of tap water in the center of each well (Table 1). The same protocol was done for 24-wells oviplate (Corning™ Incorporated Costar™ number 3526), but using 2.0 cm diameter filter paper, a 5.0 mL syringe plunger (Figure 1G–I), and 0.125 mL of tap water (Table 1).

**Figure 2:**
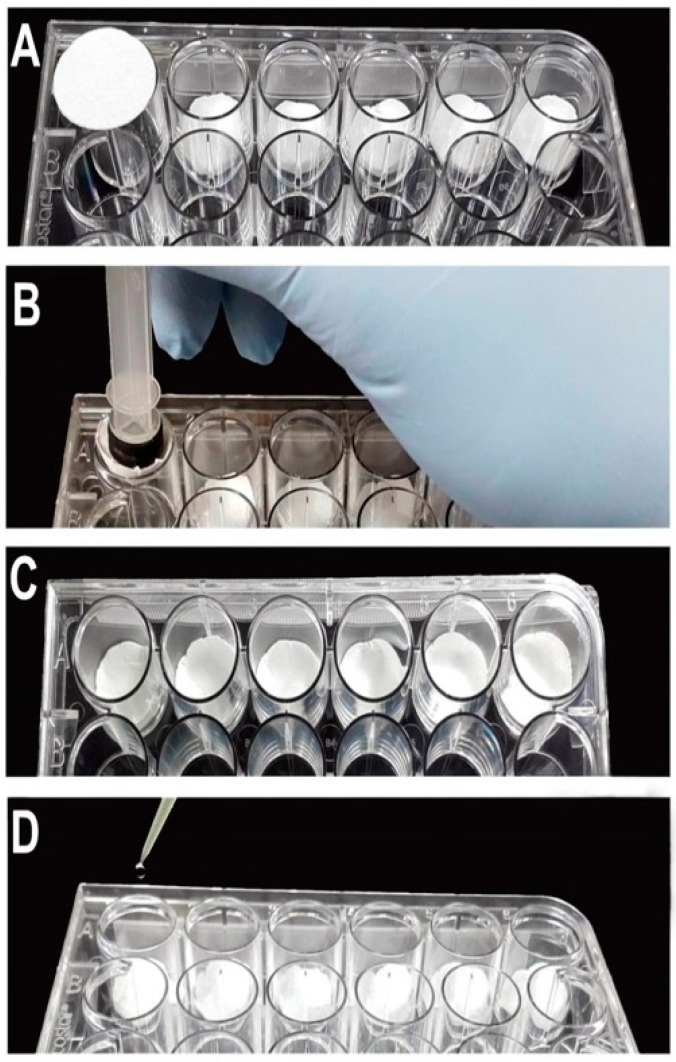
Steps to setup the oviplates using a 24-well plate. (**A**) Place the filter paper (2.0 cm diameter) on the well. (**B**) Push down the filter paper using a 5.0 mL syringe plunger. (**C**) Experimental wells with the filter paper. (**D**) Add 0.125 mL of water in the middle of the filter paper.

The plates were assembled using the same technique, but with different volumes of water and filter paper sizes according to the type of plate used (Table 1). To demonstrate the plate assembly, we used an oviplate with 24-wells (Figure 2). Initially, the filter paper was placed into the well (Figure 2A) and pushed down using the 5.0 mL syringe plunger (Figure 2B). After fitting the filter papers into all the wells (Figure 2C), 0.125 mL of water was added in the middle of each filter paper (Figure 2D).

### 2.6. Individual Oviposition Assay

**Figure 3:**
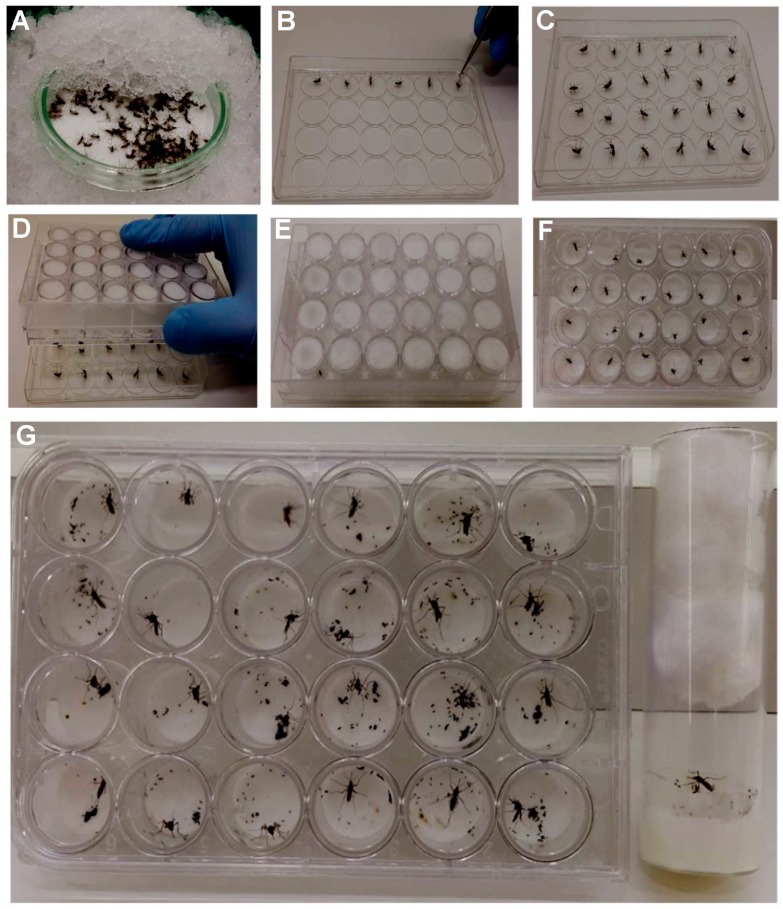
The steps to assemble the oviposition assay using the 24-well plate. (**A**) Put the anesthetized vitellogenic females on a glass petri dish over crushed ice for 15 min. (**B**) Place the females in the middle of the circle mark of the plate cover lid using forceps. (**C**) Completed lid with female mosquitoes. (**D**) Positioning the mounted plate upside down to cover the lid containing the ice-anesthetized mosquitoes. (**E**) Wait the mosquitoes to recover. (**F**) Invert the plate to the right position. (**G**) Keep the oviplate inside the insectary.

To allow all the females have completed the vitellogenesis/ovogenesis process, five-day-old PBM gravid females were anesthetized with CO_2_ and were transferred into a glass petri dish. The plate was covered with crushed ice for 15 min (Figure 3A). For oviposition in *Drosophila* vials, individual *Ae. aegypti* females were placed inside of the tubes (lying down position) until their recovery. On the other hand, for 12- and 24- well plates, the females were placed in the middle of the circle mark of the coverlid using forceps (Figure 3B,C). In both situations, females were not placed on the wet filter paper inside the well to prevent their adherence. The mounting plate was positioned upside down to cover the lid (Figure 3D) until the females recovered consciousness (Figure 3E). Finally, the plate was flipped again to the upright position (Figure 3F). Two pieces of tape were used to hold the lid on the plate. The plates and the *Drosophila* vials were kept inside the insectary for 48 h (Figure 3G). To remove the females, *Drosophila* vials and plates were put on ice until females were anesthetized (about 10 min).

### 2.7. Cost Estimation

The Fisher Scientific website (https://www.fishersci.com/us/en/home.html) was used as a reference to estimate the costs of the 12-well or 24-well plates (Corning™ Incorporated Costar™) in comparison to a *Drosophila* vial as price reference only (Fisherbrand™ *Drosophila* vials) (catalog numbers: 07-200-82, 09-761-146, and AS574, respectively). Costs are per female oviposition.

### 2.8. Fecundity Comparison between the Methods

To validate the use of 12- and 24-well cell culture plates as an oviposition method (“oviplate”), the number of eggs laid by each female was counted and compared with the number obtained from individual females laying inside the *Drosophila* vials. The oviposition assays were performed with 12 females for each device and three independent biological experiments were performed (36 females were analyzed in total).

All eggs laid on the filter paper of different oviposition methods were counted by using a Leica EZ4 stereoscope and a hand tally counter (Fisherbrand™).

### 2.9. Statistical Analysis

The average number and standard deviation of the mean were estimated for each group. The Kruskal–Wallis test (non-parametric data) followed by Dunn’s multiple comparison test were applied with a confidence interval of 95%. The analysis was performed in GraphPad Prism5 software package (Version 5.00) for Windows (San Diego, CA, USA).

## 3. Results

We hypothesize that cell culture plates would be successfully used as a device to analyze individual *Ae. aegypti* oviposition outcomes. To answer this question, a comparison was done with a common method, in this case, the *Drosophila* vial. Three parameters were evaluated: the convenience, the room/space needed, and the cost evaluation. Four premises were established to determine the best method: 1—requiring the minimum physical space to analyze individual gravid females; 2—presenting the most convenient way to assemble the device to set up the oviposition assay, 3—to be the most affordable and cost-effective method; 4—the proposed oviplate method must not provide significantly lower number of eggs (in comparison with the results obtained by individual females tested into the gold standard, *Drosophila* vials).

**Figure 4:**
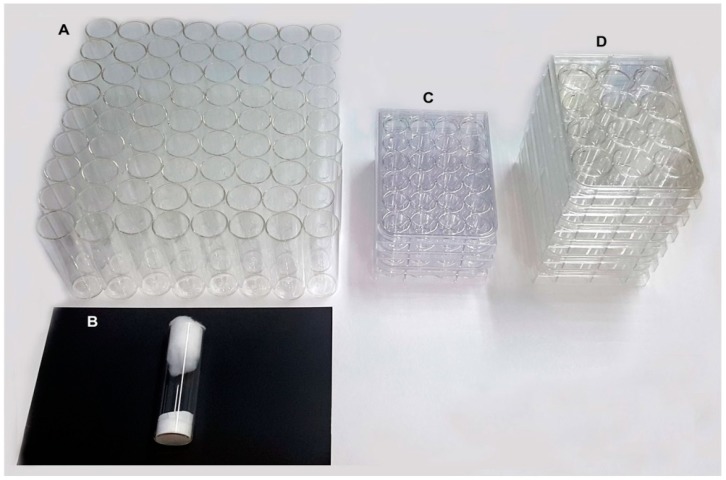
Comparison of the area used to perform oviposition experiments with *Drosophila* vials device when 72 gravid *Ae. aegypti* females are required and the proposed oviplates necessary to fit the same number of females. (**A**) The 72 *Drosophila* vials occupying 4100 cm^3^ of insectary space. (**B**) The *Drosophila* vial set up as an individual oviposition device. (**C**) Three 24-well plate occupying 700 cm^3^. (**D**) Six 12 well plate occupy 1400 cm^3^.

The room allocated to 72 gravid females was 4000 cm^3^ for *Drosophila* vials (Figure 4A,B), 1400 cm^3^ and 700 cm^3^ for 12- or 24- well plates (Figure 4C,D), respectively.

The plates were assembled using the same technique, but with different volumes of water and filter paper sizes according to the type of plate used (Table 1). To demonstrate the plate assembly, we used an oviplate with 24-wells (Figure 2). Initially the filter paper was placed into the well (Figure 2A) and pushed down using the 5.0 mL syringe plunger (Figure 2B). After fitting the filter papers into all the wells (Figure 2C), 0.125 mL of water was added in the middle of each filter paper (Figure 2D).

Once the plate is ready, gravid *Ae. aegypti* females were anesthetized and were put on a petri dish (Figure 3A). Following the anaesthetization period, they were transferred to the middle of the circle mark of the cover lid (Figure 3B,C). The mounted plate covered the lid (Figure 3D) until the females could stand up (Figure 3E), then the plate was inverted again to the right position (Figure 3F). After 48 h, *Drosophila* vials and oviplates were placed inside the crushed ice for 10 min to remove the females and for counting the eggs.

**Figure 5:**
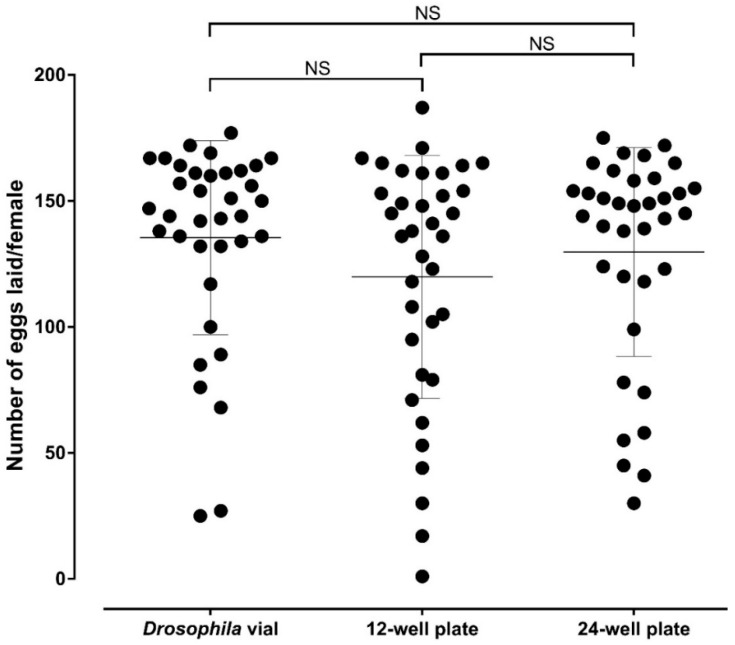
Comparison of the average number of eggs laid by female using the *Drosophila* vial (standard ovivial) and the 12- and 24-well oviplates. Five days after blood meal, *Ae. aegypti* females were individually placed in different oviposition devices. After 48 h, females were ice-anesthetized, removed from ovivials and 12- and 24-well plates and the laid eggs from each group were counted. Three biological replicates were performed, with 12 females analyzed in each group (36 females total). Each black dot represents the number of eggs laid by one female. The average number of eggs laid per female are indicated by horizontal lines and error bars are showing the standard deviation of the mean (S.D.). No significant differences were observed in the oviposition assays between the *Drosophila* vial and 12-well plate (*p* = 0.5210), *Drosophila* vial and 24-well plate (*p* > 0.9999), as well as between 12- and 24-well plates (*p* = 0.9999) by using Kruskal–Wallis test followed by Dunn’s multiple comparison test.

The average number of eggs laid (SD) per female was 135.4 (38.49) in *Drosophila* vials against 119.9 (48.2) and 129.7 (41.39) in 12- and 24-well oviplates, respectively. All females from each group deposited at least one egg. It was also possible to count the number of eggs/female in each group, an advantage of the individual ovipositon assay. There are no significant differences between the average number of eggs per female found in *Drosophila* vials in relation to the 12- (*p* = 0.5210) and 24-well (*p* > 0.9999) oviplates, as well as between both oviplates (*p* > 0.9999) (Figure 5). In addition, there is no significant differences among the three groups when each of the three independent trial was analyzed separately.

In addition to the easy assembling and comparable results, we estimated the costs for analyzing 72 females in the two methods, Oviplates and *Drosophila* vials. The average costs for six 12-well and three 24- well culture plates to perform the analysis are $22.32 and $12.24, respectively, in comparison with $102.24 for the *Drosophila* vials (Table 1). This is approximately 4.5 and 8.2 times less expensive, respectively, than the *Drosophila* vials.

## 4. Discussion

Currently, many studies associated with oviposition and fecundity in *Ae. aegypti* mosquitoes have been using tubes/*Drosophila* vials to determine reproductive changes [26,27]. With the purpose of developing an efficient individual oviposition method in *Ae. aegypti*, we have adapted 12- and 24-well cell culture plates for this type of experiment and named the assay device named an oviplate (Figure 2).

Different brands of cell-culture plates might have a lower price (considering importation, local producers, and transport), and it could even further decrease the cost of the oviposition analyses, but the Corning™ cell culture plate was selected to perform the oviplate experiments, because it presents rings on the lid that delimit each well. This feature prevents females from escaping and reaches other wells, as was observed using another brand of cell culture plates.

Different from *Drosophila* vials, the use of cotton underneath the filter paper or for closing the glass tube is not necessary for oviplates (Table 1). All oviposition assays were performed using an odorless blotting filter paper and gloves to prevent the odors influencing the oviposition behavior. Moreover, it was necessary to carefully dump the water on the filter paper (Figure 2D), preventing females from laying their eggs on the plastic wall (the same for the *Drosophila* vials).

We also adjusted the way we transfer the females to the oviplate lid. As the lid placed on ice can acquire condensed water, females can lay on the lid, which is not desirable. Instead of this, a glass Petri dish is rested on crushed ice and the CO_2_ anaesthetized females are transferred into the dish and are kept for 15 min (Figure 3A) before their allocation on the lid (Figure 3B,C). In this manner, 24 cold-anaesthetized females could be safely handled by the trained technician/user (around 5 min) (Figure 3D,F).

The oviposition time and the manner females are removed from the oviplates changes according to each experimental objective. In this study, the females were removed by putting the oviplates and *Drosophila* vials on ice for 10 min, and all females were transferred to a plastic cup with a meshed lid using the forceps. However, if the experiment requires further analyses of the offspring, it is possible to open the oviplates into an empty mosquito cage (if the experiment does require to track individuals). In this way, the eggs are not affected by the low temperature and the embryos can be further analyzed. As the *Drosophila* vials, the oviplates can be re-used. After removing the filter paper containing the eggs laid, the plates must be frozen at −20 °C for 24 h as a biological safety procedure before they can be washed with a sponge, odorless soap, and be dried at room temperature.

Moreover, although we have not tested this, mosquitoes with a similar oviposition behavior in the laboratory (*Ae. albopictus* for instance) should be suitable for laying in the oviplates as well.

## 5. Conclusions

There is no statistical difference between the number of eggs laid in *Drosophila* vials and 12- or 24-wells oviplates (Figure 5). The oviposition outcome was not altered because of the material and space variation to lay, meaning that oviplates can be used as a new, efficient, space-saving, and affordable oviposition method.

The oviplate is a quick, efficient and a cheap way to perform high-throughput oviposition assays for studying the reproductive *fitness* of *Ae. aegypti* mosquitoes.

## Figures and Tables

**Table 1:** Costs per mosquito tested, amount of cotton, filter paper diameter, and volume of water required in different methods for oviposition assays.

Oviposition Assay	Costs *	Cotton under the Paper	Cotton as a Lid	Filter Paper Diameter	Volume of Water
*Drosophila* vial (1 tube)	$1.42	0.4 g	0.5 g	2.5 cm	4.0 mL
12-well plate (1 well)	$0.31	not necessary	not necessary	2.5 cm	0.2 mL
24-well plate (1 well)	$0.17	not necessary	not necessary	2.0 cm	0.125 mL

* The estimated values are not considering cotton, filter paper, and water.
